# Complementary Feeding Practices and Nutritional Status Among Pakistani Children Aged 6–23 Months: A Pooled Analysis of Nationally Representative Surveys

**DOI:** 10.3390/nu18203389

**Published:** 2026-10-16

**Authors:** Arjumand Rizvi, Afsah Bhutta, Muhammad Sajid, Sajid B. Soofi, Zulfiqar A. Bhutta

**Affiliations:** 1 Institute for Global Health and Development, The Aga Khan University, Karachi 74800, Pakistan; 2 Centre for Global Child Health, The Hospital for Sick Children, Toronto, ON M5G 1X8, Canada; 3 Center of Excellence in Women & Child Health, The Aga Khan University, Karachi 74800, Pakistansajid.soofi@aku.edu (S.B.S.)

**Keywords:** breastfeeding, complementary feeding, non-human milk, animal milk, stunting, wasting, micronutrient deficiency, infant and young child feeding, Pakistan

## Abstract

**Background:** Milk feeding practices are a critical determinant of nutritional status in infancy, yet the relationship between specific milk feeding combinations and undernutrition remains poorly characterized in Pakistan, where animal milk use is widespread and the malnutrition burden is among the highest in South Asia. **Methods:** We pooled data on 96,736 children aged 6–23 months with valid anthropometry from 13 nationally and provincially representative cross-sectional surveys (2010–2020). Analyses were restricted to Punjab, Sindh, Khyber Pakhtunkhwa and Balochistan. Seven mutually exclusive feeding combinations were defined from 24 h dietary recall. Survey-weighted, age-stratified logistic regression (6–11 and 12–23 months) examined associations with stunting and wasting, adjusting progressively for area, household and child-level factors, with breastfeeding plus complementary feeding as the reference. Micronutrient deficiency data from NNS 2011 and NNS 2018 were analyzed descriptively. **Results:** Among infants aged 6–11 months, non-human milk (NHM) alone—predominantly unfortified buffalo or cow milk—was independently associated with elevated odds of stunting (OR: 1.54, 95% CI: 1.20–1.96) and wasting (OR: 1.86, 95% CI: 1.42–2.45) in fully adjusted models. Among children aged 12–23 months, NHM with complementary foods (NHM+CF) was independently protective against both stunting (OR: 0.73, 95% CI: 0.66–0.80) and wasting (OR: 0.80, 95% CI: 0.72–0.89). Household poverty was the strongest determinant of undernutrition in every stratum. Anemia (59.1%), ferritin deficiency (50.4%), iron deficiency anemia (33.0%) and vitamin D deficiency (63.1%) remained highly prevalent in 2018, and were highest in children receiving milk without complementary foods. In contrast, multiple micronutrient deficiency was elevated across most NHM-containing feeding groups regardless of complementary food status; after adjustment, NHM+CF and BM+NHM remained independently associated with multiple micronutrient deficiency at 12–23 months (OR: 1.35, 95% CI: 1.04–1.77 and OR: 1.52, 95% CI: 1.05–2.20, respectively). **Conclusions:** The nutritional correlates of NHM use are strongly age-dependent: adverse in the first year when it substitutes for breast milk without complementary foods, and favorable after 12 months as part of a diversified diet. Feeding interventions should be embedded within poverty-reduction frameworks. All data were cross-sectional, so these associations are not causal.

## 1. Introduction

Milk feeding practices in infancy—particularly the initiation and continuation of breastfeeding—have been the focus of many studies in Pakistan [1,2,3,4,5,6,7,8,9,10,11,12,13]. Relatively little information exists on the use of formula milk and animal milk among children 6–23 months of age, and its association with nutritional status [14]. The decision to use either formula milk or animal milk is influenced by several factors, such as the availability of breast milk, cultural customs, and the child’s specific nutritional requirements [15,16]. The World Health Organization (WHO) recommends exclusive breastfeeding for the first six months of life and continued breastfeeding, alongside adequate complementary foods, up to two years of age or beyond [17]. Breast milk remains a major source of energy, essential fatty acids and immunological protection throughout the complementary feeding period, and it is not interchangeable with other milks [17,18]. Non-human milk should be regarded as an alternative or supplement only when breastfeeding is not possible or breast milk is insufficient. For such infants aged 6–11 months, formula milk or full-fat animal milk may be used. For young children aged 12–23 months, WHO favors animal milk over follow-up formula, as it provides essential nutrients such as calcium, riboflavin, potassium, phosphorus, magnesium, zinc, and protein and stimulates production of insulin-like growth factor-1 (IGF-1), which supports bone development and growth [17].

Malnutrition remains a major public health challenge among young children in low- and middle-income countries, contributing to an estimated 45% of deaths in children under five years of age [19]. Pakistan has one of the highest stunting rates in South Asia and it continues to experience a high burden of child undernutrition, with stunting affecting approximately 40% of children under five years of age [3,20,21], with marked disparities by residence, province and wealth [3]. Micronutrient deficiencies are also widespread among children under five; the prevalence of iron deficiency anemia (IDA), vitamin A deficiency (VAD) and iodine deficiency (IoD) are 29%, 52% and 40% respectively, and vitamin D deficiency affects 62.7% of children aged 6–59 months [3].

Determinants of undernutrition and micronutrient deficiencies in Pakistan include poverty, food security, access to healthcare and nutrition services, dietary practices and environmental conditions [22,23,24]. Financial constraints can limit the ability of families to afford a varied and nutritious diet [25,26].

Milk provides essential nutrients such as calcium and high-quality proteins that support growth after six months of age [17,27]. The pathways through which milk feeding influences child growth are multiple and biologically plausible. Breast milk provides immunological protection and reduces the burden of infection, a proximal driver of both wasting and stunting. Non-human milks, when introduced during the complementary feeding window (6–23 months), may substitute for or supplement breast milk and complementary foods, affecting energy density and micronutrient intake. The type of non-human milk (animal versus formula milk) has substantially different nutritional profiles, particularly regarding iron, zinc, and vitamin C bioavailability, with implications for micronutrient status. However, the direction and magnitude of these associations in the Pakistani context, characterized by widespread animal milk use (predominantly buffalo milk), suboptimal dietary diversity, and high infection burden, remain insufficiently characterized.

We conducted an analysis of milk feeding and complementary feeding practices among young children aged 6–23 months, the WHO complementary feeding window, using nationally representative surveys (the Pakistan Demographic and Health Surveys (PDHS), National Nutrition Surveys (NNS), and Multiple Indicator Cluster Surveys (MICS)) from 2010 to 2020. Pooling surveys with harmonized infant and young child feeding (IYCF) indicators provides an opportunity to examine trends and associations with adequate statistical power.

The specific objectives of this analysis were: (i) to describe trends in milk feeding practices and complementary diet patterns among children aged 6–23 months across Pakistan from 2010 to 2020; (ii) to examine the association between milk feeding combinations and the risk of stunting and wasting; and (iii) to explore the relationship between feeding patterns and micronutrient deficiencies using biochemical data available from the National Nutrition Surveys.

## 2. Materials and Methods

### 2.1. Data Sources

In this study, we utilized all available microdata from nationally representative surveys conducted over the past 15 years, spanning from 2010 to 2020. These surveys encompassed the Pakistan Demographic and Health Surveys (PDHS), Multiple Indicator Cluster Surveys (MICS), and serial National Nutrition Surveys (NNS). The DHS and NNS were administered nationwide with subnational power, while the MICS were conducted within respective provinces at various time points with district-level specificity. These nationally representative cross-sectional surveys used the sampling frame developed by the Pakistan Bureau of Statistics (PBS) and followed a two-stage, stratified cluster sampling approach. The detailed sampling methodology of these surveys can be found in the respective reports [1,2,3,4,5,6,7,8,9,10,11,12,13]. PDHS [2,4] and NNS 2011 [1] have provincial specificity while NNS 2018 [3] and MICS [5,6,7,8,9,10,11,12,13] have district-level representativeness. All surveys included standardized data on feeding practices and anthropometric measurements, while biochemical micronutrient data were available only in NNS 2011 [1] and NNS 2018 [3].

Anthropometric measurements were taken by trained teams using standardized equipment and procedures in each survey. Height-for-age and weight-for-height z-scores were derived using the WHO Child Growth Standards [28]. Biologically implausible anthropometric values (beyond −5 SD for WHZ and −6 SD for HAZ) were treated as missing and were not included in the analysis (Table S1).

All data sources were harmonized prior to data pooling, and PSU identifiers were made unique across the 13 surveys. Feeding indicators, outcomes and covariates were mapped to a common classification framework. All PDHS, MICS and NNS 2018 collected 24 h dietary recall data for children using the WHO standardized module; however, minor differences exist in the prompting structure and food groupings across surveys. Only NNS 2011 employed a quantitative 24 h dietary assessment tool. Food categories were recoded to ensure consistency across surveys. These differences are acknowledged as a limitation.

Micronutrient status indicators were available exclusively from the National Nutrition Survey 2011 and NNS 2018. In both rounds, hemoglobin was measured in the field on capillary blood using a HemoCue machine. Venous blood samples were processed, and serum was transported under cold chain to the Nutrition Research Lab, Aga Khan University (AKU) laboratory, Karachi, for analysis. Serum ferritin, C-reactive protein (CRP) and α1-acid glycoprotein (AGP) were measured by immunoturbidimetric assay, retinol by high-performance liquid chromatography (HPLC), zinc by atomic absorption spectrophotometry, and 25-hydroxyvitamin D by chemiluminescence immunoassay. All biochemical assessments followed standardized laboratory protocols with external quality assurance. Hemoglobin values were adjusted for altitude. Ferritin, vitamin A and zinc concentrations were adjusted for the effect of inflammation using CRP and AGP [29,30]. Hemoglobin, ferritin, vitamin A, vitamin D and zinc deficiency were defined as hemoglobin < 11.0 g/dL, inflammation-adjusted ferritin < 12 µg/L, serum retinol < 0.70 µmol/L, serum zinc < 60 µg/dL and 25-hydroxyvitamin D < 20 ng/mL. Multiple micronutrient deficiency was defined as deficiency in three or more of these five indicators. The feeding–micronutrient analyses are therefore restricted to the NNS subsample.

### 2.2. Study Variables and Definitions

#### 2.2.1. Outcomes

The primary outcome of this study in relation to feeding patterns was childhood stunting, defined as the child’s height-for-age z-score of less than −2 SD. The study also considered several secondary outcomes, including wasting (characterized by a weight-for-height z-score of less than −2 SD), anemia, iron deficiency anemia, and the presence of multiple micronutrient deficiencies (MMN) in association with milk consumption patterns. Within the scope of MMN, the analysis encompasses deficiencies in hemoglobin, ferritin, vitamin A, zinc, and vitamin D. Data on biochemical indicators were available only from the NNS.

#### 2.2.2. Exposure

Milk feeding practices constituted the primary exposure of this study. These practices were assessed through structured questions relying on a 24 h dietary recall. Since children consumed various milk types either individually or in conjunction with complementary foods (CF), a discrete classification of feeding combinations was formulated. This classification encompassed breast milk (BM), formula milk (FM), and animal milk (AM). However, it is noteworthy that the consumption of FM was consistently lower than that of AM at all time points, resulting in several combinations with small cell sizes (Figure S1). For analytic stability, a merged category named ‘non-human milk (NHM)’ was developed, effectively including the utilization of either FM or AM.

The reference category for feeding exposure in all models was BM+CF (breastfeeding with complementary feeding) in line with WHO-recommended practices for children aged 6–23 months and represents the expected optimal group against which deviations in risk can be meaningfully compared.

#### 2.2.3. Time

As the MICS were conducted in the four provinces at different time points, we grouped the surveys by round and combined them with nationally representative DHS and NNS survey data to illustrate time trends. The time variable is not equally spaced because of variations in survey timing.

#### 2.2.4. Age Stratification

Because feeding practices vary substantially by child age—with breastfeeding-dominant combinations concentrated among younger infants and complementary-food-dominant combinations prevailing in older children—the analysis was stratified into two age groups (6–11 months and 12–23 months) to allow a more plausible and unconfounded assessment of the exposure–outcome relationship within each developmental stage (Table S2).

#### 2.2.5. Covariates

Covariates included in the multivariable models were household socio-demographic indicators (wealth quintile, flooring, roof and wall materials, and number of rooms used for sleeping), drinking-water source and sanitation, urban/rural residence, province, maternal education, child age, sex and size at birth, and the standard IYCF indicators of minimum dietary diversity, minimum meal frequency and minimum acceptable diet.

### 2.3. Statistical Analysis

To ensure consistent geographical coverage across surveys, only the four provinces of Punjab, Sindh, Khyber Pakhtunkhwa and Balochistan were included; Islamabad Capital Territory, Gilgit-Baltistan, Azad Jammu and Kashmir and the former FATA were excluded. Descriptive statistics were reported in terms of frequency and percentage. Given consistent feeding patterns and malnutrition trends observed in the descriptive analysis, a pooled analysis was conducted across all surveys. Survey year was included as a categorical covariate in the pooled models to account for secular trends. To assess whether associations were homogeneous across survey platforms, an interaction term between survey type (NNS vs. PDHS vs. MICS) and feeding category was assessed in preliminary models. No statistically significant interaction was detected (*p* = 0.07 for 6–11 months, 0.20 for 12–23 months), supporting the validity of pooling.

For the multivariate analysis, logistic regression was employed to assess the impact of exposure on the outcomes. To evaluate this impact, three models were developed. The first model examined the association between the outcome and the exposure, adjusted only for child age and survey round. The second model incorporated the outcome–exposure relationship while adjusting for household-level factors, including demographic parameters and sanitation practices. Additionally, the third model incorporated individual-level factors including maternal education, child age, sex, size at birth, and IYCF practices, in addition to the factors included in the second model. Further, the analysis was stratified by age. Survey round was included as a categorical fixed effect in all models to account for secular trends in stunting and wasting prevalence across the study period. Results are presented as odds ratios (ORs) with 95% confidence intervals (CIs). No adjustment was made for multiple comparisons in primary analysis; a sensitivity analysis was performed using Benjamini–Hochberg false discovery rate (FDR) correction [31] for the primary outcome on the full adjusted model for the two age strata. Complete-case analysis was performed for each model.

All analyses considered the survey design, including clustering and sampling weights. Within-survey sampling weights were rescaled to ensure that each survey contributed proportionally to the pooled estimates in proportion to its own unweighted sample size rather than to the size of the population it represents. This rescaling was implemented by dividing each observation’s survey weight by the sum of weights within its survey and multiplying by the unweighted sample size of that survey, following standard practice for pooled analysis of nationally representative surveys. Analyses were conducted in Stata version 18.0 (Stata Corp LLC, College Station, TX, USA) using the ‘svy’ suite with subpopulation estimation.

## 3. Results

Overall, 128,049 children aged 0–23 months were identified across the 13 surveys, of whom 96,736 were aged 6–23 months (Table 1). After exclusion of records with missing feeding data (*n* = 5800) or missing or biologically implausible HAZ (*n* = 10,910) or WHZ (*n* = 10,152) data, 80,682 (6–11: 27,263; 12–23: 53,419) and 81,330 (6–11: 27,338; 12–23: 53,992) children aged 6–23 months formed the analytic sample for stunting and wasting respectively (Table S1). The pooled sample includes five survey time periods from 2010-11 to 2018-20, with Punjab contributing the largest share of observations across all rounds. Balochistan had the fewest survey time points available, with MICS rounds only conducted in 2010 and 2019-20.

### 3.1. Demographic Characteristics

The socio-demographic characteristics of survey participants depict consistent patterns. The data reveals that the distribution of housing, access to improved water sources and sanitation, and educational status remains remarkably constant over the observed period. Moreover, the age and gender distribution of children across all surveys also exhibit a high level of consistency, underscoring that the data effectively captures key population characteristics (Table 2).

### 3.2. Malnutrition Status

**Figure 1:**
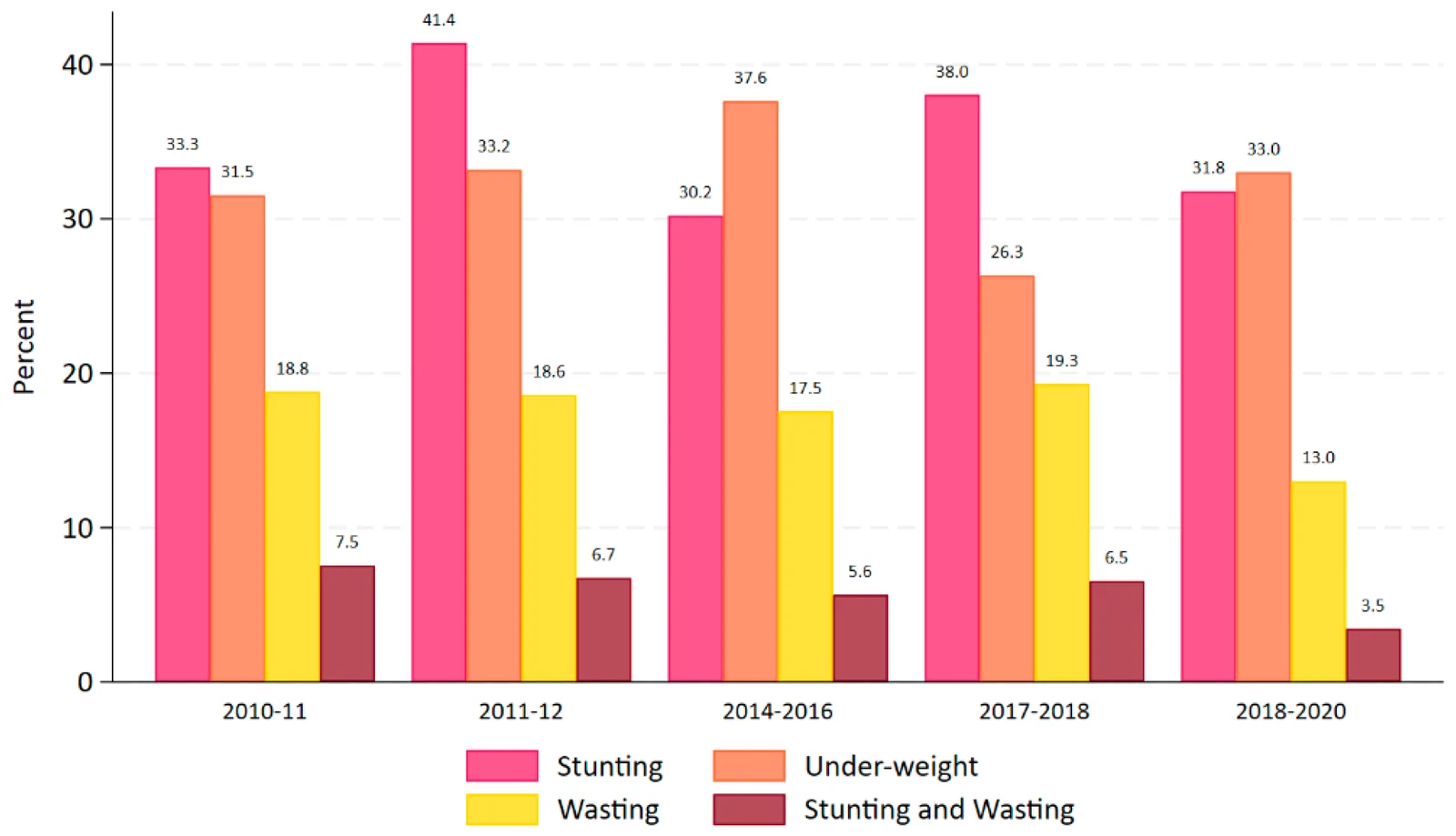
Prevalence of stunting, wasting, underweight and concurrent wasting and stunting among children aged 6–23 months, by survey period.

Among the various forms of malnutrition, the burden of stunting stands out as the highest in nearly all surveys. Approximately one-third of children aged 6–23 months were stunted with the highest prevalence (41.4%) noted in 2011-12. The prevalence of underweight mirrored this trend, as approximately 32% of the children were underweight. The wasting rate was also consistent up until 2018, with roughly 20% of children in this age group wasted; this proportion fell to 13% in 2018-19. Similarly, the prevalence of concurrent malnutrition (both wasting and stunting) was around 6.6% until 2018, but it decreased to 3.5% in 2018-19. Regarding the provinces, Sindh and Balochistan bear the heaviest burden of malnutrition (Figure 1).

### 3.3. Feeding Trends and Patterns by Age

Feeding combination distribution showed moderate variation across survey rounds, reflecting both genuine temporal shifts and survey-related differences. BM+NHM+CF was the most prevalent combination in two of the five periods (29.8–31.0%); NHM+CF predominated in 2010-11 (27.4%) and BM+CF in 2011-12 (31.6%). Minimum dietary diversity, unavailable for the 2010-11 surveys, showed a gradual but consistently upward trend, improving from 6.0% in 2011-12 to 14.2% in 2018-20 while remaining critically low throughout the decade. The proportion of children meeting minimum meal frequency showed considerable variation (27.9–57.2%), which likely reflects methodological differences across survey platforms rather than true secular change and should be interpreted with caution (Table 2).

**Figure 2:**
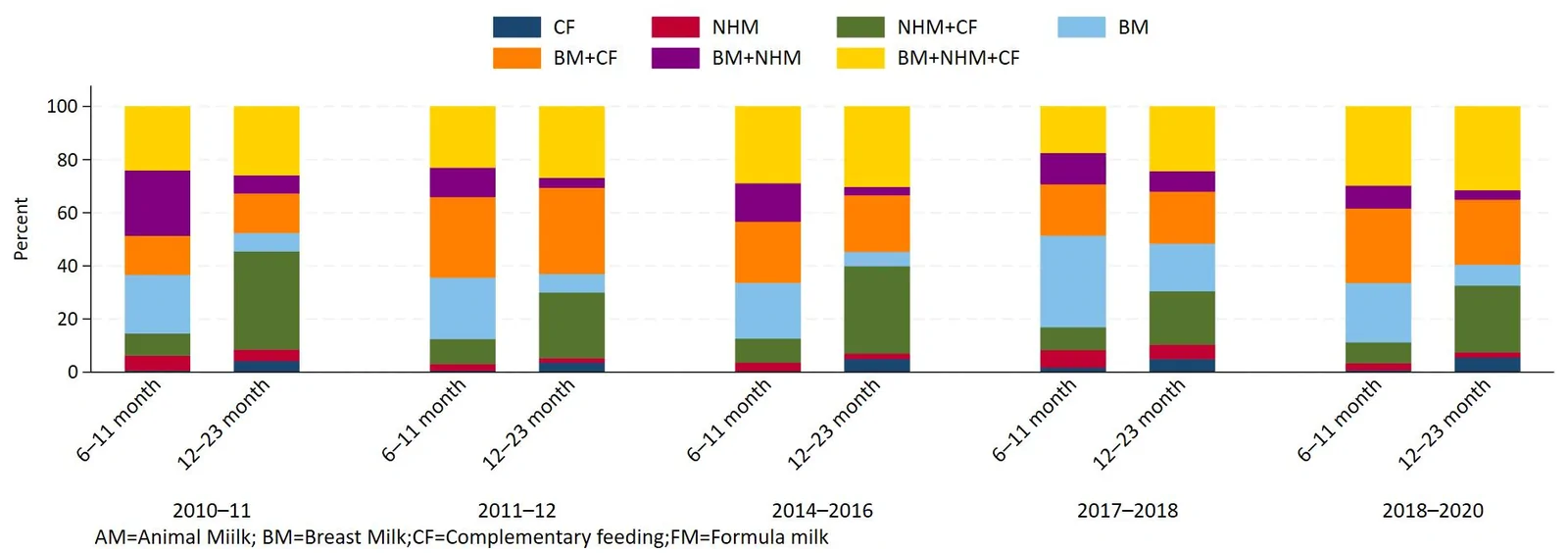
Distribution of the seven mutually exclusive feeding combinations among children aged 6–11 and 12–23 months, by survey period.

Feeding practices differed markedly between the two age strata (Figure 2; Table S2). Among children aged 6–11 months, breastfeeding in any form was near-universal (86.5%), with BM+NHM+CF (25.2%), BM alone (24.3%) and BM+CF (22.5%) the three most common combinations. Animal milk was received by 47.6% and formula milk by 10.3% of children in this age group. Among children aged 12–23 months, breastfeeding declined to 62.5%, while complementary food consumption rose to 79.9%. NHM+CF (28.5%) and BM+NHM+CF (28.1%) were the predominant combinations, and animal milk use increased to 61.5%. These contrasting distributions confirm the strong age-patterning of feeding practices and underpin the rationale for stratified analysis (Table S2; Figure S1).

### 3.4. Micronutrient Deficiencies

Among children aged 6–23 months in the two National Nutrition Surveys, the most notable reduction was in zinc deficiency, which fell from 37.2% in 2011 to 18.7% in 2018, followed by anemia, which fell from 70.2% to 59.1% over the same period although it remained very high. On the other hand, the prevalence of vitamin D deficiency increased from 39.4% in 2011 to 63.1% in 2018. The burden of ferritin deficiency was also remarkably high; 47% of children had ferritin deficiency in 2011, increasing slightly to 50.4% in 2018. Approximately one-third of children aged 6–23 months had three or more concurrent deficiencies among hemoglobin, ferritin, vitamin A, zinc, and vitamin D (Table S3).

### 3.5. Association Between Stunting and Feeding Practices

**Figure 3:**
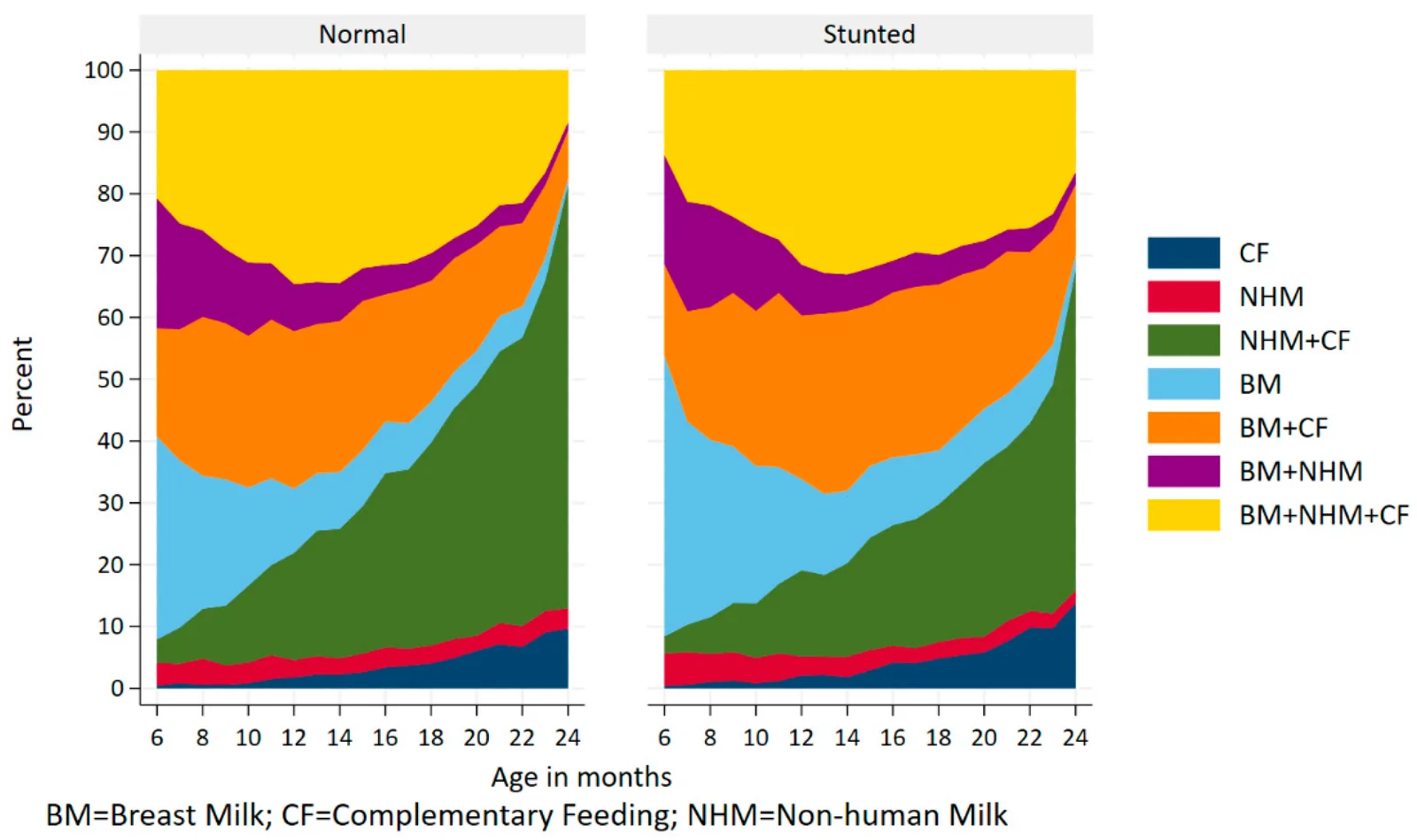
Distribution of milk feeding combinations by stunting status, children aged 6–23 months.

The exploratory analysis revealed that feeding patterns varied by age for stunted and non-stunted children. BM consumption was higher among stunted children specifically in younger ages (Figure 3). Among infants aged 6–11 months, stunting prevalence was highest in those receiving BM alone (29.0%) and NHM alone (30.0%), and lowest in those receiving BM+NHM+CF (20.8%) and NHM+CF (20.9%). Among children aged 12–23 months, stunting prevalence was lowest among those receiving NHM+CF (32.1%), followed by NHM alone (35.9%), and highest among those receiving BM alone (45.6%) and CF alone (44.0%) (Table S4).

#### 3.5.1. Age 6–11 Months

In the model adjusted only for child age and survey round (Model 1), NHM alone and BM alone were each independently associated with significantly elevated odds of stunting relative to the reference category of BM+CF: NHM (OR: 1.43, 95% CI: 1.20–1.70, *p* < 0.001) and BM (OR: 1.41, 95% CI: 1.28–1.56, *p* < 0.001). BM+NHM was also associated with modestly elevated stunting odds (OR: 1.15, 95% CI: 1.02–1.29, *p* = 0.023), while BM+NHM+CF and NHM+CF showed a small protective association. CF alone did not differ significantly from BM+CF at this age (Table S5).

After adjustment for area and household-level factors (Model 2), the associations for NHM (OR: 1.53, 95% CI: 1.25–1.88) and BM alone (OR: 1.39, 95% CI: 1.24–1.55) persisted and were marginally strengthened, as did BM+NHM (OR: 1.18, 95% CI 1.04–1.34). The protective signal for BM+NHM+CF and NHM+CF was attenuated, suggesting these associations were confounded by socioeconomic factors (Table S5).

In the fully adjusted model (Model 3), NHM alone remained the feeding category most robustly and consistently associated with increased stunting odds (OR: 1.54, 95% CI: 1.20–1.96, *p* = 0.001). BM alone retained a significant elevated association (OR: 1.32, 95% CI: 1.15–1.51, *p* < 0.001), as did BM+NHM (OR: 1.19, 95% CI: 1.02–1.39, *p* = 0.023). Among covariates, Balochistan (OR: 2.31, 95% CI: 1.85–2.88) and Sindh (OR: 1.52, 95% CI: 1.36–1.71) showed markedly elevated stunting odds relative to Punjab. Poverty showed a strong gradient: children in the poorest wealth quintile had nearly double the stunting odds compared to the richest quintile. Maternal illiteracy (OR: 1.35, 95% CI: 1.21–1.51), male sex (OR: 1.17, 95% CI: 1.07–1.28), low birth weight or small size at birth (OR: 1.60, 95% CI: 1.44–1.77), and suboptimal meal frequency (OR: 1.12, 95% CI 1.01–1.25) were each independently associated with stunting in this age group (Table 3).

#### 3.5.2. Age 12–23 Months

The pattern of stunting and feeding associations among children aged 12–23 months differed substantially from that observed in younger infants. In the model adjusted only for child age and survey round (Model 1), NHM+CF was associated with the greatest reduction in stunting odds (OR: 0.54, 95% CI: 0.50–0.57, *p* < 0.001), followed by NHM alone (OR: 0.68, 95% CI: 0.59–0.79, *p* < 0.001), BM+NHM+CF (OR: 0.83, 95% CI: 0.78–0.89, *p* < 0.001), CF alone (OR: 0.86, 95% CI: 0.77–0.96, *p* = 0.005) and BM+NHM (OR: 0.89, 95% CI: 0.80–0.99, *p* = 0.040). BM alone showed modestly elevated odds (OR: 1.10, 95% CI: 1.01–1.21, *p* = 0.030) (Table S5).

After household adjustment (Model 2), the protective associations for NHM+CF (OR: 0.67, 95% CI: 0.62–0.71), CF alone (OR: 0.86, 95% CI: 0.77–0.96), and NHM alone (OR: 0.80, 95% CI: 0.68–0.95) remained statistically significant, while BM+NHM+CF and BM+NHM were attenuated (Table S4). In the fully adjusted model (Model 3), NHM+CF (OR: 0.73, 95% CI: 0.66–0.80, *p* < 0.001) and CF alone (OR: 0.84, 95% CI: 0.73–0.96, *p* = 0.010) were retained as protective feeding combinations. BM alone, NHM alone, BM+NHM, and BM+NHM+CF were all non-significant after full adjustment, indicating that these associations were largely attributable to confounding by socioeconomic and child-level factors.

Among covariates, poverty exerted the strongest influence; children in the poorest quintile had 2.47 times the odds of stunting compared to the richest (OR: 2.47, 95% CI: 2.16–2.83), with a clear graded relationship across all quintiles. Balochistan (OR: 2.04, 95% CI: 1.74–2.38), Sindh (OR: 1.65, 95% CI: 1.53–1.78), and KP (OR: 1.26, 95% CI: 1.16–1.38) all showed significantly elevated stunting odds relative to Punjab. Maternal illiteracy (OR: 1.37, 95% CI: 1.28–1.47), poor dietary diversity (OR: 1.30, 95% CI: 1.18–1.42), minimum acceptable diet not met (OR: 1.30, 95% CI: 1.14–1.47), low birth weight (OR: 1.41, 95% CI: 1.31–1.52), and male sex (OR: 1.07, 95% CI: 1.01–1.13) were each independently associated with stunting (Table 3).

The associations survive after adjusting for FDR for the both age stratum.

### 3.6. Association Between Wasting and Feeding Practices

Among infants aged 6–11 months, wasting prevalence was highest in those receiving NHM alone (24.2%), and lowest in those receiving BM+CF (14.1%) and CF alone (14.8%). Among children aged 12–23 months, wasting prevalence was highest in those receiving BM+NHM (20.4%), and lowest in those receiving NHM+CF (15.0%) (Table S4).

NHM alone showed the strongest and most consistent association with wasting across all three models in children aged 6–11 months. In the model adjusted for child age and survey round (Model 1), NHM was associated with substantially elevated wasting odds (OR: 1.67, 95% CI: 1.37–2.04, *p* < 0.001). After household adjustment (Model 2), this association strengthened further (OR: 1.99, 95% CI 1.59–2.48, *p* < 0.001) (Table S6). In the fully adjusted model (Model 3), NHM alone remained robustly associated with wasting (OR: 1.86, 95% CI: 1.42–2.45, *p* < 0.001), making it the only feeding category with a significant and consistent signal across all three models in this age group. BM+NHM was also associated with elevated wasting odds in Models 1 and 2 (OR: 1.21 and 1.24 respectively), with the association persisting after full adjustment (OR: 1.26, 95% CI: 1.02–1.54, *p* = 0.030). BM+NHM+CF showed a borderline significance in Model 3 (OR: 1.18, 95% CI: 1.01–1.38, *p* = 0.038) (Table 4).

In contrast to younger infants, the only significant feeding association with wasting among children aged 12–23 months across all three models was for NHM+CF, which showed a consistent protective association: Model 1 (OR: 0.75, 95% CI: 0.69–0.81, *p* < 0.001), Model 2 (OR: 0.80, 95% CI: 0.74–0.87, *p* < 0.001), and Model 3 (OR: 0.80, 95% CI: 0.72–0.89, *p* < 0.001). No other feeding category associated with wasting in this age group (Table S6).

Among covariates, maternal illiteracy, male sex, low birth weight, poor socioeconomic status, and residence in Sindh were each associated with a higher likelihood of wasting in both age bands. Residence in Khyber Pakhtunkhwa was associated with higher wasting odds at 6–11 months (OR 1.22, 95% CI 1.04–1.44) but with lower odds at 12–23 months (OR 0.81, 95% CI 0.71–0.92). Poor sanitation was associated with increased wasting odds (OR 1.25, 95% CI: 1.14–1.37) at age 12–23 months (Table 4).

### 3.7. Association Between Micronutrient Deficiencies and Feeding Practices

**Figure 4:**
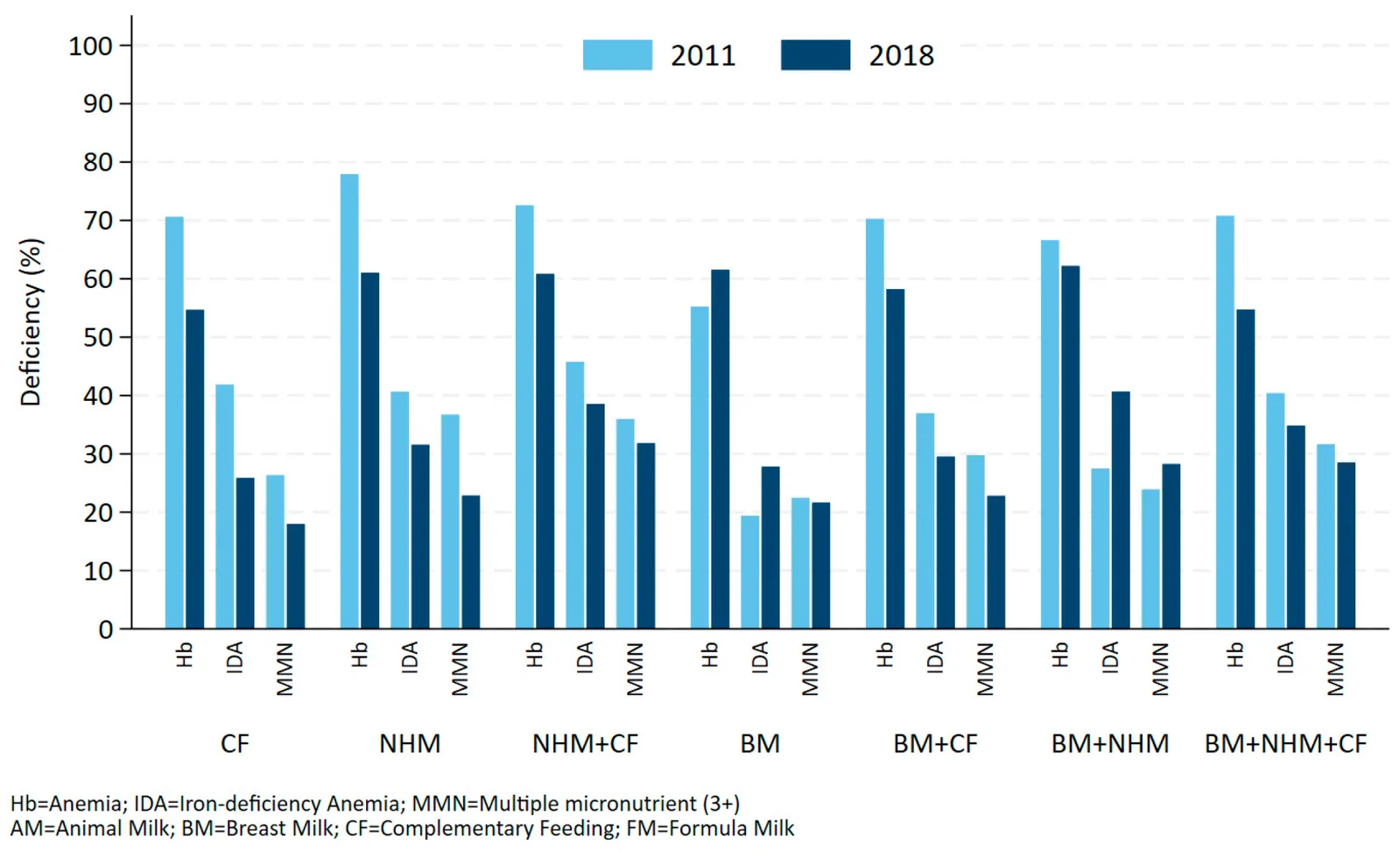
Proportion of children aged 6–23 months with micronutrient deficiencies by feeding combination, NNS 2011 and NNS 2018.

Exploratory analysis of biochemical data from NNS 2011 and NNS 2018 revealed a consistent pattern of elevated micronutrient deficiency burden among children receiving milk without complementary foods. In 2018, the highest proportions of anemia and ferritin deficiency were observed in children receiving BM+NHM without complementary foods, consistent with the combined low iron content of breast milk in later lactation and unfortified animal milk. Children in NHM alone and BM+NHM groups showed persistently elevated deficiency proportions across both survey years. Conversely, children in the BM+NHM+CF and NHM+CF groups—those receiving both milk and complementary foods—showed comparatively lower deficiency burdens. Multiple micronutrient deficiencies were highest in the NHM+CF, BM+NHM and BM+NHM+CF groups (Figure 4).

In the model for multiple micronutrient deficiencies (Table 5), no feeding category was significantly associated with MMN deficiency among children aged 6–11 months. Among children aged 12–23 months, NHM+CF (OR: 1.35, 95% CI: 1.04–1.77, *p* = 0.027) and BM+NHM (OR: 1.52, 95% CI: 1.05–2.20, *p* = 0.027) were each significantly associated with increased odds of MMN deficiency. BM+NHM+CF showed a similarly elevated but non-significant point estimate (OR: 1.23, 95% CI: 0.99–1.54, *p* = 0.065); CF alone and BM alone were not significant. This association is consistent in all models (Table S7).

## 4. Discussion

This pooled analysis of 96,736 children aged 6–23 months drawn from 13 nationally and provincially representative surveys across Pakistan (2010–2020) provides comprehensive evidence on the relationship between milk feeding practices and child undernutrition in Pakistan. Three major findings emerge. First, non-human milk consumed alone without complementary foods is independently associated with elevated odds of both stunting and wasting among infants aged 6–11 months, with this association robust to full adjustment for household socioeconomic, maternal, and child-level confounders. Second, among children aged 12–23 months, NHM consumed with complementary foods is independently protective against both stunting and wasting. Third, across both age strata and both outcomes, poverty and illiteracy are the dominant determinants of undernutrition, with feeding practices substantially attenuated after socioeconomic adjustment, particularly in the older age group. Together, these findings have direct implications for how Pakistan designs and targets its infant and young child feeding strategy.

### 4.1. Age-Dependent Consequences of Non-Human Milk Use

The finding that NHM alone is the feeding category most robustly associated with both stunting (OR: 1.54, 95% CI: 1.20–1.96) and wasting (OR: 1.86, 95% CI: 1.42–2.45) in the 6–11-month age group, with associations strengthening on progressive covariate adjustment, provides grounds for concern about the widespread early introduction of unfortified animal milk in Pakistan. Buffalo milk, which constitutes the predominant non-human milk source in the country, has high energy and fat content but provides very little iron and vitamin C, both essential for iron absorption and immune competence in early infancy [32]. When introduced as a breast milk substitute, animal milk displaces immunologically active breast milk, imposes a renal solute load on immature kidneys, and fails to meet micronutrient requirements at a developmental window when iron demands, growth velocity, and infection susceptibility are simultaneously at their peak [33]. The elevated wasting odds in the NHM-alone group reinforce that the risk is not limited to long-term growth faltering but extends to acute nutritional depletion, likely mediated through increased infection burden and reduced immune defense. Descriptive comparison of formula milk and animal milk as separate (overlapping) exposures (Table S4) showed closely similar stunting and wasting prevalence in both age groups suggesting the pooled NHM category is not being driven disproportionately by one milk type, though this comparison is unadjusted and formula milk use in these settings is uncommon.

Comparable findings have been reported in other South Asian and sub-Saharan African settings, where early introduction of unmodified cow or buffalo milk has been associated with iron deficiency anemia, growth faltering and failing to achieve developmental milestones [34,35,36]. The persistence of this association after adjustment for poverty and sanitation in the present analysis is notable: unlike many feeding–growth associations that attenuate substantially in fully adjusted models, the NHM-alone effect remained robust. While this pattern is consistent with a genuine nutritional pathway, residual or unmeasured confounding and differential misclassification of feeding status cannot be excluded, and the persistence of the association after adjustment does not by itself establish causality.

Among older children aged 12–23 months, the pattern inverts. NHM with complementary foods exhibits consistent protective association with both stunting (OR: 0.73, 95% CI: 0.66–0.80) and wasting (OR: 0.80, 95% CI: 0.72–0.89) in fully adjusted models. This is biologically coherent: after 12 months, renal solute load tolerance improves, gut maturity allows more effective nutrient absorption, and the child’s requirement for dietary iron can increasingly be met through diversified complementary foods rather than relying solely on milk [37]. The energy and protein density of full-fat buffalo milk may actively support catch-up growth in this age group when consumed alongside iron-providing solid foods. However, this association should not be interpreted as nutritional superiority of non-human milk over breast milk. The attenuation of ORs in Model 1 to Model 3 indicates substantial confounding, and residual confounding by unmeasured household advantage (livestock ownership, food security, quantity of complementary foods) and maternal factors (employment, nutritional status) is likely. Reverse causation, the mechanism described in Section 4.2 for younger children, may persist into the second year and inflate malnutrition. Moreover, NHM+BF+CF showed no association with growth, and NHM+CF was associated with higher odds of multiple micronutrient deficiency. This age-dependent pattern supports age-specific guidance on milk feeding rather than uniform messages, while continued breastfeeding with adequate complementary foods remains the recommended practice in the second year of life [17,18].

### 4.2. Reverse Causation and the Breastfeeding Paradox

Breastfeeding alone was associated with elevated stunting odds in the 6–11-month age group (OR: 1.32, 95% CI: 1.15–1.51) even after full adjustment, a finding that merits careful interpretation rather than causal attribution. In cross-sectional surveys, children who are growing poorly for reasons unrelated to feeding including intrauterine growth restriction, recurrent infection, or genetic predisposition are more likely to remain predominantly or exclusively breastfed at ages when their peers have already transitioned to diversified diets, because caregivers often intensify breastfeeding in response to perceived poor growth and because sick or slow-growing children may be less capable of accepting solid foods [18]. This phenomenon, sometimes described as the weanling’s dilemma [38] in observational literature, generates a spurious positive association between breastfeeding and poor growth in cross-sectional data that does not reflect a causal nutritional pathway. Figure 3 is consistent with this interpretation. Maternal nutritional status offers a further potential explanation. Maternal dietary inadequacies and micronutrient deficiencies can influence breast-milk concentrations of certain micronutrients, including vitamin A, several B vitamins, and iodine, although the extent of this influence varies by nutrient [39,40]. Undernourished mothers and children may also share the same food-insecure households, potentially confounding the observed association between breastfeeding and stunting. These findings should not be construed as evidence against breastfeeding, which retains strong and well-established immunological and developmental benefits, particularly in the first six months of life.

### 4.3. Micronutrient Deficiencies and the Role of Feeding

Children receiving BM+NHM without complementary foods had the highest proportions of anemia and ferritin deficiency in NNS 2018, a finding explicable by the compounded iron inadequacy of late-lactation breast milk and unfortified buffalo milk consumed in the absence of iron-containing solid foods. This group represents a particularly high-risk category that does not consistently emerge from standard IYCF indicator frameworks, yet carries a micronutrient burden arguably greater than children receiving neither breast milk nor complementary foods. After adjustment for socioeconomic and child-level confounders, NHM+CF (OR 1.35, 95% CI 1.04–1.77) and BM+NHM (OR 1.52, 95% CI 1.05–2.20) remained independently associated with multiple micronutrient deficiency at 12–23 months, indicating this burden is not fully explained by poverty or maternal education. The coexistence of lower odds of stunting and wasting with higher odds of multiple micronutrient deficiency in the NHM+CF group is biologically plausible. Animal milk supplies the energy and protein that support growth, but it is low in iron and vitamin D, inhibits non-heme iron absorption, and may displace iron-rich foods [33]. Adequate growth should therefore not be taken as evidence of adequate micronutrient status. Targeted supplementation or provision of fortified complementary foods for children in this feeding pattern warrants consideration as a program priority [41].

### 4.4. Socioeconomic and Child-Level Determinants of Undernutrition

Household poverty was the strongest and most consistent determinant of both stunting and wasting across all models and age strata. Fully adjusted stunting odds in the poorest wealth quintile were 1.92 (95% CI: 1.55–2.37) at 6–11 months and 2.47 (95% CI: 2.16–2.83) at 12–23 months, with a clear gradient across quintiles and comparable wealth effects on wasting. The minimal attenuation of these associations between household-adjusted and fully adjusted models indicates that the poverty–undernutrition relationship operates largely upstream of dietary diversity and meal frequency factors which are themselves expressions of food access rather than independent behavioral choices and aligns with a substantial body of South Asian evidence demonstrating that feeding-focused interventions alone have limited population-level impact without concurrent improvements in household economic conditions and food security [42]. At the child and maternal level, low birth weight was among the strongest predictors of both stunting and wasting across all age groups and models, underscoring the well-established continuity between fetal growth restriction and postnatal undernutrition and reinforcing the case for antenatal nutrition investment including iron-folate supplementation and management of maternal undernutrition as upstream interventions in the causal chain [43]. Maternal illiteracy showed consistent independent associations with both outcomes, reflecting the pathways through which education shapes feeding knowledge, health-seeking behavior, and dietary quality [44]. Male sex was associated with elevated stunting and wasting odds in both age strata, consistent with the well-documented biological vulnerability of male infants in South Asia [45]. Poor dietary diversity was associated with both stunting and wasting at 12–23 months, and failure to meet the minimum acceptable diet with stunting only, whereas neither indicator was associated with either outcome at 6–11 months, a pattern that reflects the greater measurement validity and nutritional significance of dietary diversity indicators in the second year of life, when complementary foods constitute a larger share of total energy and nutrient intake. That dietary diversity retained an independent association with growth outcomes even after adjustment for wealth confirms that diet quality carries nutritional effects beyond its role as a socioeconomic proxy [46].

### 4.5. Strengths and Limitations

The principal strengths of this analysis are its large, pooled sample, its temporal coverage using multiple complementary survey platforms, and its use of three progressively adjusted regression models that enable transparent examination of confounding pathways. Age stratification was driven empirically by the observed distribution of feeding practices rather than by an a priori decision. Rescaling of within-survey sampling weights prior to pooling ensures proportional survey contribution, and the use of the subpopulation estimation approach preserves the full complex survey design for variance calculation in all stratified models. The triangulation of findings across PDHS, MICS, and NNS platforms each with independent sampling frames strengthens confidence in the consistency of observed patterns.

Several limitations must be acknowledged. All surveys employed cross-sectional designs, precluding causal inference or establishing temporality in the feeding–growth relationship. Single 24 h dietary recalls are subject to within-person variation and may misclassify habitual intake. Children who receive complementary foods or non-human milk intermittently may be misclassified, and the same applies to the dietary diversity, meal frequency and acceptable diet indicators used as covariates. Non-differential misclassification would generally bias associations towards the null, so the elevated odds observed for NHM alone at 6–11 months may be conservative. However, misclassification may also be differential: a child who is unwell on the recall day may refuse solid foods and be recorded as receiving milk only, which would inflate associations with wasting in particular. Repeated recalls or multi-day records, which were not available in these surveys, would be needed to characterize habitual feeding patterns [47]. The merging of formula and animal milk into a single NHM category, necessitated by small formula milk cell sizes, may mask heterogeneous effects given the substantially different micronutrient profiles of these two milk types. Minor differences in IYCF module structure across PDHS, MICS4, and later MICS rounds limit strict comparability of certain indicators over time. Because stunting and wasting are common, the odds ratios reported here overstate the corresponding prevalence ratios and should be compared cautiously with studies reporting risk or prevalence ratios. Residual confounding is likely, in particular from unmeasured morbidity, birth order and birth interval, maternal stature and body mass index, and household food insecurity; wealth indices are survey-specific and only approximately comparable across platforms. Islamabad Capital Territory, Gilgit-Baltistan, Azad Jammu and Kashmir and the former FATA were excluded, so findings are not generalizable to those areas. Finally, reverse causation cannot be excluded for any of the feeding–growth associations reported. The multiple micronutrient deficiency indicator counted five deficiencies equally, although their clinical significance differs. Two components (anemia and low ferritin) share an iron pathway, and vitamin D deficiency was present in almost two-thirds of children in 2018, so the composite is driven largely by iron and vitamin D status. Equal weighting may therefore overstate the public health burden it represents. Future studies should explore weighted composite indices, for example by severity or attributable disease burden, or data-driven approaches such as latent class analysis. Additionally, the survey rounds differed in sample size across provinces (Table 1), which may affect the precision and comparability of prevalence estimates across time.

### 4.6. Policy Implications

These findings carry clear and actionable implications for Pakistan’s infant and young child feeding policy. Evidence from systematic reviews indicates that complementary feeding interventions delivered alone produce small improvements in linear growth, and that larger gains require concurrent action on household food security, income, women’s education and environmental health [48,49]. Our findings should therefore not shift policy emphasis towards non-human milk. Interventions to improve maternal nutrition during pregnancy and lactation, protect and support breastfeeding, and ensure adequate complementary feeding should be delivered together, with guidance on non-human milk reserved for children who are not breastfed or receive insufficient breast milk [17,50].

For infants aged 6–11 months, the data provides strong empirical grounds for discouraging unfortified animal milk as a breast milk substitute and for prioritizing breastfeeding continuation alongside timely introduction of micronutrient-rich complementary foods. Community-level behavior change communication targeting early introduction of animal milk deserves intensification, particularly in Balochistan and Sindh where malnutrition burden is highest and NHM use without complementary feeding is prevalent. For children aged 12–23 months who are no longer breastfed, the data support inclusion of animal milk as part of a diversified diet; however, the high iron and vitamin D deficiency burden in this group underscores the need for targeted micronutrient supplementation or provision of fortified complementary foods for non-breastfed toddlers [42]. The consistent association of poverty across all models highlights the importance of linking infant and young child feeding (IYCF) programs with social protection mechanisms. In addition, strategies that enhance economic empowerment and reduce financial constraints are crucial for achieving sustainable development and lasting nutritional gains.

## 5. Conclusions

In this pooled analysis of 13 nationally and provincially representative Pakistani surveys, the association between non-human milk and child undernutrition was strongly age-dependent: in the fully adjusted model non-human milk fed alone in the first year was associated with higher odds of stunting and wasting, whereas non-human milk fed alongside complementary foods in the second year was associated with lower odds of both. Household poverty, maternal illiteracy and low birth weight were the dominant determinants of undernutrition throughout. Age-specific feeding guidance is therefore needed, but is unlikely to deliver population-level gains. Household poverty, food insecurity, maternal education, low birth weight and water and sanitation operate upstream of feeding behavior and constrain what families can feed their children. Feeding guidance should therefore be delivered alongside social protection, maternal nutrition and water and sanitation programs.

## Figures and Tables

**Table 1:** Data sources and number of children aged 6–23 months included in the analysis, by province, survey platform, survey round and age group.

	Punjab		Sindh		KP		Balochistan		Overall	
	6–11 Months	12–23 Months	6–11 Months	12–23 Months	6–11 Months	12–23 Months	6–11 Months	12–23 Months	6–11 Months	12–23 Months
**NNS**										
2011	1350	2081	734	887	252	352	223	319	2559	3639
2018	2005	4100	1063	2088	626	1317	714	1485	4408	8990
**PDHS**										
2012-13	294	789	195	448	226	450	147	289	862	1976
2017-18	280	725	159	419	178	417	124	250	741	1811
**MICS**										
Round 4 (2010)	NA	NA	NA	NA	NA	NA	556	1523	556	1523
Round 4 (2011)	7029	14,081	NA	NA	NA	NA	NA	NA	7029	14,081
Round 5 (2014)	3005	5814	1772	3343	NA	NA	NA	NA	4777	9157
Round 5 (2016)	NA	NA	NA	NA	2155	4306	NA	NA	2155	4306
Round 6 (2018)	3326	7881	1453	3131	NA	NA	NA	NA	4779	11,012
Round 6 (2019)	NA	NA	NA	NA	2231	4608	1456	4080	3687	8688
Total	17,289	35,471	5376	10,316	5668	11,450	3220	7946	31,553	65,183

NA time points at which no MICS was conducted. MICS rounds are shown by year. This study includes two NNS, two PDHS and 9 MICS resulting in 13 surveys conducted between 2010 and 2020 in four provinces of Pakistan.

**Table 2:** Demographic characteristics and feeding patterns of children aged 6–23 months, by survey period (weighted percentages).

	Time					
	2010-11	2011-12	2014–2016	2017–2018	2018–2020	Total
*N*	23,189	9036	20,395	15,950	28,166	96,736
**Flooring material**						
Natural	38.7%	45.1%	45.3%	34.6%	47.6%	43.5%
Rudimentary	6.0%	0.5%	0.1%	0.5%	1.0%	2.0%
Finished	55.3%	54.4%	54.6%	64.9%	51.4%	54.5%
**Material for roof**						
Natural	1.4%	6.4%	1.7%	4.3%	6.1%	3.7%
Rudimentary	24.9%	8.9%	21.7%	13.6%	19.0%	19.6%
Finished	73.7%	84.7%	76.6%	82.2%	74.9%	76.6%
**Material for wall**						
Natural	23.4%	12.9%	17.7%	10.8%	24.5%	20.3%
Rudimentary	7.4%	8.1%	4.4%	9.8%	8.7%	7.4%
Finished	69.3%	79.1%	77.9%	79.4%	66.9%	72.4%
**Rooms for sleeping**						
One	36.0%	41.9%	32.9%	33.9%	29.3%	33.6%
Two	33.2%	33.8%	31.1%	35.5%	30.6%	32.1%
Three or more	30.7%	24.3%	36.0%	30.6%	40.1%	34.2%
**Main source of drinking water**						
Unimproved water	5.1%	4.4%	4.0%	6.5%	6.1%	5.2%
Improved water	94.9%	95.6%	96.0%	93.5%	93.9%	94.8%
**Improved sanitation**						
Unimproved sanitation	29.6%	22.0%	23.5%	14.4%	25.7%	24.9%
Improved sanitation	70.4%	78.0%	76.5%	85.6%	74.3%	75.1%
**Maternal education**						
Illiterate	51.9%	55.5%	52.3%	50.7%	57.8%	53.8%
Literate	48.1%	44.5%	47.7%	49.3%	42.2%	46.2%
**Age group**						
6–11 months	33.4%	37.6%	34.3%	32.5%	29.9%	32.8%
12–23 months	66.6%	62.4%	65.7%	67.5%	70.1%	67.2%
**Gender**						
Male	51.3%	51.6%	51.1%	51.0%	51.5%	51.3%
Female	48.7%	48.4%	48.9%	49.0%	48.5%	48.7%
**Feeding**						
CF	3.0%	2.4%	3.3%	3.9%	3.9%	3.4%
NHM	4.8%	2.1%	2.5%	5.8%	2.2%	3.5%
NHM+CF	27.4%	18.9%	24.7%	16.3%	19.4%	21.8%
BM	12.0%	13.1%	10.7%	23.4%	12.6%	14.2%
BM+CF	14.8%	31.6%	21.9%	19.5%	25.6%	21.7%
BM+NHM	12.7%	6.6%	7.0%	9.0%	5.2%	8.2%
BM+NHM+CF	25.4%	25.4%	29.8%	22.1%	31.0%	27.1%
**Dietary diversity**						
No	-	94.0%	88.4%	87.1%	85.8%	87.9%
Yes	-	6.0%	11.6%	12.9%	14.2%	12.1%
**Minimum meal frequency**						
No	62.9%	43.8%	42.8%	72.1%	45.8%	53.7%
Yes	37.1%	56.2%	57.2%	27.9%	54.2%	46.3%
**Minimum acceptable diet**						
No	-	93.7%	93.3%	95.9%	93.6%	94.1%
Yes	-	6.3%	6.7%	4.1%	6.4%	5.9%

BM = breast milk; CF = complementary feeding; NHM = non-human milk (formula + animal milk). Data shown as weighted percentages.

**Table 3:** Association between feeding category and stunting status among children aged 6–23 months: fully adjusted (Model 3) weighted logistic regression.

	Stunting: 6–11 Months			Stunting: 12–23 Months		
	OR	95% CI	*p*-Value	OR	95% CI	*p*-Value
Feeding = CF	0.94	0.54, 1.64	0.836	0.84	0.73, 0.96	0.010
Feeding = NHM	1.54	1.20, 1.96	0.001	0.92	0.75, 1.13	0.418
Feeding = NHM+CF	1.10	0.89, 1.34	0.375	0.73	0.66, 0.80	<0.0001
Feeding = BM	1.32	1.15, 1.51	<0.0001	1.11	0.98, 1.27	0.102
Feeding = BM+CF	Ref.			Ref.		
Feeding = BM+NHM	1.19	1.02, 1.39	0.023	0.93	0.78, 1.10	0.387
Feeding = BM+NHM+CF	1.01	0.89, 1.15	0.841	0.96	0.89, 1.04	0.341
Region = Urban	Ref.			Ref.		
Region = Rural	1.03	0.90, 1.17	0.705	0.99	0.91, 1.07	0.795
Province = Punjab	Ref.			Ref.		
Province = Sindh	1.52	1.36, 1.71	<0.0001	1.65	1.53, 1.78	<0.0001
Province = KP	0.95	0.84, 1.08	0.460	1.26	1.16, 1.38	<0.0001
Province = Balochistan	2.31	1.85, 2.88	0.000	2.04	1.74, 2.38	<0.0001
Main source of drinking water = Unimproved	0.89	0.73, 1.09	0.247	0.89	0.77, 1.02	0.082
Main source of drinking water = Improved	Ref.			Ref.		
Main source of sanitation = Unimproved	1.17	1.04, 1.31	0.007	1.18	1.09, 1.28	<0.0001
Main source of sanitation = Improved	Ref.			Ref.		
Wealth index (quintiles) = Poorest	1.92	1.55, 2.37	<0.0001	2.47	2.16, 2.83	<0.0001
Wealth index (quintiles) = Second	1.49	1.22, 1.82	<0.0001	2.08	1.84, 2.36	<0.0001
Wealth index (quintiles) = Middle	1.20	0.99, 1.45	0.068	1.58	1.41, 1.78	<0.0001
Wealth index (quintiles) = Fourth	1.10	0.91, 1.33	0.319	1.33	1.19, 1.48	<0.0001
Wealth index (quintiles) = Richest	Ref.			Ref.		
Maternal education = Illiterate	1.35	1.21, 1.51	<0.0001	1.37	1.28, 1.47	<0.0001
Maternal education = Literate	Ref.			Ref.		
Gender = Male	1.17	1.07, 1.28	<0.0001	1.07	1.01, 1.13	0.026
Gender = Female	Ref.			Ref.		
Status at birth = Normal	Ref.			Ref.		
Status at birth = LBW/smaller at birth	1.60	1.44, 1.77	<0.0001	1.41	1.31, 1.52	<0.0001
Dietary diversity = No	1.22	0.99, 1.52	0.068	1.30	1.18, 1.42	<0.0001
Dietary diversity = Yes	Ref.			Ref.		
Minimum meal frequency = No	1.12	1.01, 1.25	0.034	1.02	0.95, 1.08	0.650
Minimum meal frequency = Yes	Ref.			Ref.		
Minimum acceptable diet = No	1.39	0.90, 2.14	0.136	1.30	1.14, 1.47	<0.0001
Minimum acceptable diet = Yes	Ref.			Ref.		

BM = breast milk; CF = complementary feeding; NHM = non-human milk (formula + animal milk). Estimates are adjusted for area, household, maternal education, child age, and survey round and account for the complex survey design (stratification, clustering and rescaled sampling weights).

**Table 4:** Association between feeding category and wasting status among children aged 6–23 months: fully adjusted (Model 3) weighted logistic regression.

	Wasting: 6–11 Months			Wasting: 12–23 Months		
	OR	95% CI	*p*-Value	OR	95% CI	*p*-Value
Feeding = CF	0.76	0.38, 1.49	0.423	0.88	0.74, 1.04	0.140
Feeding = NHM	1.86	1.42, 2.45	0.000	0.93	0.73, 1.18	0.539
Feeding = NHM+CF	1.19	0.95, 1.51	0.135	0.80	0.72, 0.89	0.000
Feeding = BM	1.10	0.93, 1.30	0.289	0.97	0.83, 1.14	0.734
Feeding = BM+CF	Ref.			Ref.		
Feeding = BM+NHM	1.26	1.02, 1.54	0.030	1.15	0.94, 1.41	0.182
Feeding = BM+NHM+CF	1.18	1.01, 1.38	0.038	1.00	0.91, 1.10	0.986
Region = Urban	Ref.			Ref.		
Region = Rural	0.89	0.77, 1.03	0.111	0.91	0.81, 1.03	0.128
Province = Punjab	Ref.			Ref.		
Province = Sindh	1.21	1.05, 1.39	0.007	1.19	1.08, 1.31	0.000
Province = KP	1.22	1.04, 1.44	0.012	0.81	0.71, 0.92	0.001
Province = Balochistan	0.85	0.67, 1.09	0.192	0.86	0.72, 1.03	0.096
Main source of drinking water = Unimproved	1.05	0.85, 1.31	0.636	1.10	0.93, 1.30	0.276
Main source of drinking water = Improved	Ref.			Ref.		
Main source of sanitation = Unimproved	1.11	0.96, 1.28	0.149	1.25	1.14, 1.37	0.000
Main source of sanitation = Improved	Ref.			Ref.		
Wealth index (quintiles) = Poorest	1.29	1.02, 1.63	0.037	1.34	1.10, 1.64	0.004
Wealth index (quintiles) = Second	1.25	1.01, 1.55	0.044	1.15	0.94, 1.39	0.168
Wealth index (quintiles) = Middle	1.16	0.95, 1.43	0.153	1.06	0.89, 1.26	0.542
Wealth index (quintiles) = Fourth	1.06	0.86, 1.31	0.583	0.99	0.86, 1.16	0.947
Wealth index (quintiles) = Richest	Ref.			Ref.		
Maternal education = Illiterate	1.31	1.15, 1.50	<0.0001	1.11	1.02, 1.21	0.019
Maternal education = Literate	Ref.			Ref.		
Gender = Male	1.37	1.23, 1.53	<0.0001	1.26	1.18, 1.36	<0.0001
Gender = Female	Ref.			Ref.		
Status at birth = Normal	Ref.			Ref.		
Status at birth = LBW/smaller at birth	1.35	1.20, 1.53	<0.0001	1.37	1.27, 1.49	<0.0001
Dietary diversity = No	1.04	0.80, 1.35	0.766	1.23	1.10, 1.37	<0.0001
Dietary diversity = Yes	Ref.			Ref.		
Minimum meal frequency = No	0.92	0.80, 1.04	0.185	0.96	0.88, 1.04	0.273
Minimum meal frequency = Yes	Ref.			Ref.		
Minimum acceptable diet = No	1.13	0.70, 1.84	0.614	1.06	0.91, 1.23	0.481
Minimum acceptable diet = Yes	Ref.			Ref.		

BM = breast milk; CF = complementary feeding; NHM = non-human milk (formula + animal milk). Estimates are adjusted for area, household, maternal education, child age, and survey round and account for the complex survey design (stratification, clustering and rescaled sampling weights).

**Table 5:** Association between feeding category and multiple micronutrient deficiencies among children aged 6–23 months: fully adjusted (Model 3) weighted logistic regression.

	6–11 Months			12–23 Months		
	OR	95% CI	*p*-Value	OR	95% CI	*p*-Value
Feeding = CF	1.20	0.38, 3.80	0.758	0.77	0.53, 1.13	0.187
Feeding = NHM	1.26	0.73, 2.19	0.404	0.94	0.58, 1.52	0.791
Feeding = NHM+CF	1.32	0.79, 2.20	0.297	1.35	1.04, 1.77	0.027
Feeding = BM	0.91	0.64, 1.30	0.614	0.88	0.66, 1.17	0.380
Feeding = BM+CF	Ref.			Ref.		
Feeding = BM+NHM	0.80	0.53, 1.23	0.312	1.52	1.05, 2.20	0.027
Feeding = BM+NHM+CF	1.11	0.77, 1.61	0.572	1.23	0.99, 1.54	0.065
Region = Urban	Ref.			Ref.		
Region = Rural	0.93	0.68, 1.27	0.635	0.91	0.74, 1.11	0.338
Province = Punjab	Ref.			Ref.		
Province = Sindh	0.89	0.66, 1.19	0.434	0.77	0.62, 0.94	0.011
Province = KP	0.59	0.37, 0.94	0.026	0.76	0.58, 1.00	0.048
Province = Balochistan	0.55	0.32, 0.94	0.029	0.59	0.42, 0.83	0.002
Main source of drinking water = Unimproved	1.11	0.67, 1.84	0.683	0.60	0.39, 0.91	0.016
Main source of drinking water = Improved	Ref.			Ref.		
Main source of sanitation = Unimproved	1.02	0.69, 1.50	0.921	1.02	0.77, 1.36	0.878
Main source of sanitation = Improved	Ref.			Ref.		
Wealth index (quintiles) = Poorest	1.52	0.91, 2.52	0.109	1.10	0.76, 1.60	0.602
Wealth index (quintiles) = Second	1.21	0.78, 1.88	0.391	1.01	0.74, 1.38	0.943
Wealth index (quintiles) = Middle	0.97	0.65, 1.46	0.893	0.99	0.76, 1.28	0.916
Wealth index (quintiles) = Fourth	0.96	0.65, 1.41	0.833	1.13	0.88, 1.45	0.335
Wealth index (quintiles) = Richest	Ref.			Ref.		
Maternal education = Illiterate	0.85	0.65, 1.10	0.216	0.88	0.72, 1.07	0.206
Maternal education = Literate	Ref.			Ref.		
Gender = Male	1.07	0.85, 1.34	0.585	1.04	0.88, 1.22	0.664
Gender = Female	Ref.			Ref.		
Status at birth = Normal	Ref.			Ref.		
Status at birth = LBW/smaller at birth	1.05	0.76, 1.44	0.782	1.13	0.92, 1.39	0.241
Dietary diversity = No	1.33	0.56, 3.16	0.516	1.26	0.91, 1.76	0.164
Dietary diversity = Yes	Ref.			Ref.		
Minimum meal frequency = No	0.84	0.61, 1.15	0.275	1.12	0.90, 1.37	0.307
Minimum meal frequency = Yes	Ref.			Ref.		
Minimum acceptable diet = No	1.80	0.73, 4.40	0.199	0.98	0.68, 1.43	0.930
Minimum acceptable diet = Yes	Ref.			Ref.		

BM = breast milk; CF = complementary feeding; NHM = non-human milk (formula + animal milk). Estimates are adjusted for area, household, maternal education, child age, and survey round and account for the complex survey design (stratification, clustering and rescaled sampling weights).

## Data Availability

PDHS datasets are available at https://dhsprogram.com and MICS datasets at https://mics.unicef.org/surveys (accessed on 10 August 2026), both on registration and approval. NNS datasets and the Stata do-files used to derive the feeding categories and fit the models are available from the corresponding author on reasonable request.

## Supplementary Materials

The following supporting information can be downloaded at https://www.mdpi.com/article/10.3390/nu18203389/s1. Table S1: Participant’s flow and analytical sample size; Table S2. Feeding practices among children aged 6–23 months, by age group (weighted percentages, pooled across 13 surveys, 2010–2020); Table S3: Distribution of micronutrient deficiencies over time from NNS 2011 and NNS 2018 surveys; Table S4. Distribution of stunting and Wasting by feeding categories among children aged 6–23 months, by age group (weighted percentages, pooled across 13 surveys, 2010–2020); Table S5. Association between feeding category and stunting among children aged 6–23 months: survey-weighted logistic regression; Table S6. Association between feeding category and wasting among children aged 6–23 months: survey-weighted logistic regression; Table S7. Association between feeding category and multiple micronutrient deficiencies among children aged 6–23 months: survey-weighted logistic regression; Table S8: Unadjusted and adjusted *p*-values for model 3 for stunting outcome; Figure S1. Feeding combinations among children aged 6–11 and 12–23 months, by survey period.

## Abbreviations

AM, animal milk; BM, breast milk; CF, complementary feeding; CI, confidence interval; FM, formula milk; HAZ, height-for-age z-score; IDA, iron deficiency anemia; IYCF, infant and young child feeding; KP, Khyber Pakhtunkhwa; LBW, low birth weight; MAD, minimum acceptable diet; MDD, minimum dietary diversity; MICS, Multiple Indicator Cluster Survey; MMF, minimum meal frequency; MMN, multiple micronutrient deficiency; NHM, non-human milk; NNS, National Nutrition Survey; OR, odds ratio; PDHS, Pakistan Demographic and Health Survey; VAD, vitamin A deficiency; WHZ, weight-for-height z-score; WHO, World Health Organization.
