# Supplementary material for: Complementary Feeding Practices and Nutritional Status Among Pakistani Children Aged 6–23 Months: A Pooled Analysis of Nationally Representative Surveys
Source: Nutrients. 2026 Oct 16;18(20):3389. doi: 10.3390/nu18203389 (PMC13708651; doi:10.3390/nu18203389)
Supplement: Supplementary file 1 [file nutrients-18-03389-s001.zip › nutrients-4578008-supplementary.pdf]

Supplementary Materials

Table S1: Participant's flow and analytical sample size

Step	Starting N	Excluded (n)	Reason for Exclusion	Resulting N
1	133849	5800	Belongs to FATA, AJK, or Gilgit-Baltistan	**128049**
2	128049	31,313	Children aged 0–5 months	**96736** (restricting to 6-23 months)
3	96736	5800	Feeding data missing/incomplete	**90936**
4a	90936	10254	Missing/flagged HAZ	**80682** (6-11m:27263;12-23m:53419) (analytical sample for HAZ)
4b	90936	9606	Missing/flagged WHZ	**81330** (6-11m:27338;12-23m:53992) (analytical sample for WHZ)
**Micronutrient Sub-sample**				
				**6926** (6-11 m:2235; 12-23m:4691)

Note: *Steps 4a and 4b are parallel branches derived from the same base sample (N = 90,936)- one exclusion path for the HAZ analytical sample and one for the WHZ analytical sample rather than sequential exclusions.*

Table S2. Feeding practices among children aged 6–23 months, by age group (weighted percentages, pooled across 13 surveys, 2010–2020).

	Age groups		
	6-11 months	12-23 months	Total
N	30557	60379	90936
**Overlapping feeding categories**			
BM	86.5%	62.5%	70.4%
FM	10.3%	8.5%	9.1%
AM	47.6%	61.5%	56.8%
CF	57.0%	79.9%	72.4%
**Feeding combination**			
CF	0.8%	4.8%	3.4%
NHM	4.2%	3.2%	3.5%
NHM+CF	8.6%	28.5%	21.8%
BM	24.3%	9.0%	14.2%
BM+CF	22.5%	21.3%	21.7%
BM+NHM	14.5%	5.0%	8.2%

BM+NHM+CF	25.2%	28.1%	27.1%

AM=Animal milk; BM=Breast Milk; CF=Complementary feeding; FM=Formula milk; NHM=Non-human milk

*NHM = 'AM' and/or 'FM'. The overlapping feeding categories (BM, FM, AM, CF) are not mutually exclusive and therefore do not sum to 100%; the seven feeding combinations below are mutually exclusive and exhaustive.

Table S3: Distribution of micronutrient deficiencies over time from NNS 2011 and NNS 2018 surveys

	year	
	**2011**	**2018**
N	3317	3609
**Anemia**		
No	29.8%	40.9%
Hb<11 g/dl	70.2%	59.1%
**Ferritin Deficiency**		
No	53.0%	49.6%
Deficient<12 ng/dl	47.0%	50.4%
**Iron deficiency anemia**		
No	61.1%	67.0%
Yes	38.9%	33.0%
**Vitamin A deficiency**		
Normal	79.7%	88.3%
Deficient<0.35 mmol/L	20.3%	11.7%
**Zinc Deficiency**		
No	62.8%	81.3%
Deficient<60 mg/dl	37.2%	18.7%
**Vitamin D deficiency**		
Otherwise	60.6%	36.9%
Deficient <20 ng/ml	39.4%	63.1%
**Multiple micronutrient deficiencies***		
<3	69.7%	74.4%
>=3	30.3%	25.6%

* Any of haemoglobin, ferritin, vitamin A, zinc, and vitamin D deficiency

Data shown weighted percentages

Table S4. Distribution of stunting and Wasting by feeding categories among children aged 6–23 months, by age group (weighted percentages, pooled across 13 surveys, 2010–2020).

	Stunting				Wasting			
	**6-11 month (N=27263)**		**12-23 month (N=53419)**		**6-11 month (N=27338)**		**12-23 month (N=53992)**	
	**N**	**%**	**N**	**%**	**N**	**%**	**N**	**%**
**Discrete Feeding Combinations**								
CF	220	25.5	2,516	44.0	222	14.8	2,542	17.5
NHM	1046	30.0	1551	35.9	1052	24.2	1555	18.7
NHM+CF	2248	20.9	15427	32.1	2256	16.6	15560	15.0
BM	6580	29.0	4447	45.6	6595	17.0	4512	19.4
BM+CF	6226	23.1	11617	43.7	6241	14.1	11752	18.6
BM+NHM	3951	24.3	2574	40.2	3943	18.4	2611	20.4
BM+NHM+CF	6992	20.8	15287	38.8	7029	15.3	15460	17.8
**Overlapping feeding categories**								
BM	23749	24.2	33925	41.3	23808	15.9	34335	18.5
FM	2542	22.9	4218	35.2	2559	16.0	4296	15.7
AM	12811	22.6	32905	35.6	12855	17.2	33230	16.7
CF	15686	21.9	44847	37.8	15748	14.9	45314	16.9

AM=Animal milk; BM=Breast Milk; CF=Complementary feeding; FM=Formula milk; NHM=Non-human milk *NHM = 'AM' and/or 'FM'.

Data shown weighted percentages

Figure S1. Feeding combinations among children aged 6–11 and 12–23 months, by survey period.

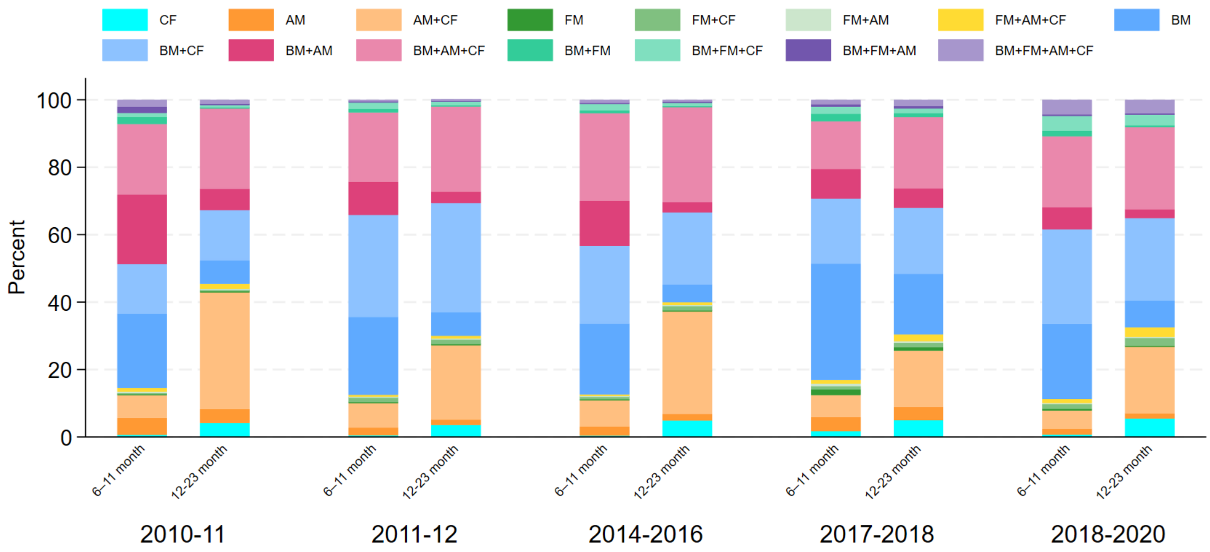

Table S5. Association between feeding category and stunting among children aged 6–23 months: survey-weighted logistic regression

	Stunting: 6-11 month						Stunting :12-23 month					
	Model-1			Model-2			Model-1			Model-2		
	OR	95% CI	p-value	OR	95% CI	p-value	OR	95% CI	p-value	OR	95% CI	p-value
Feeding=CF	1.03	0.71, 1.50	0.864	0.96	0.61, 1.53	0.878	0.86	0.77, 0.96	0.005	0.86	0.77, 0.96	0.009
Feeding=NHM	1.43	1.20, 1.70	0.000	1.53	1.25, 1.88	0.000	0.68	0.59, 0.79	0.000	0.80	0.68, 0.95	0.012
Feeding=NHM+CF	0.84	0.72, 0.98	0.023	0.98	0.84, 1.16	0.834	0.54	0.50, 0.57	0.000	0.67	0.62, 0.71	0.000
Feeding=BM	1.41	1.28, 1.56	0.000	1.39	1.24, 1.55	0.000	1.10	1.01, 1.21	0.030	1.11	1.00, 1.23	0.052
Feeding=BM+CF	***Ref.***			***Ref.***			***Ref.***			***Ref.***		
Feeding=BM+NHM	1.15	1.02, 1.29	0.023	1.18	1.04, 1.34	0.010	0.89	0.80, 0.99	0.040	0.95	0.84, 1.08	0.452
Feeding=BM+NHM+CF	0.89	0.81, 0.99	0.028	0.93	0.84, 1.03	0.182	0.83	0.78, 0.89	0.000	0.94	0.88, 1.01	0.084
Region=Urban				***Ref.***						***Ref.***		
Region=Rural				0.93	0.84, 1.03	0.182				0.94	0.87, 1.00	0.058
Province=Punjab				***Ref.***						***Ref.***		
Province=Sindh				1.46	1.31, 1.63	0.000				1.60	1.49, 1.72	0.000
Province=KP				0.95	0.85, 1.07	0.419				1.24	1.15, 1.35	0.000
Province=Balochistan				1.75	1.46, 2.09	0.000				1.49	1.32, 1.69	0.000
Main Source of drinking water=unimproved				0.91	0.76, 1.08	0.264				0.82	0.73, 0.92	0.001
Main Source of drinking water=improved				***Ref.***						***Ref.***		
Main Source of sanitation =unimproved				1.23	1.11, 1.35	0.000				1.17	1.10, 1.25	0.000
Main Source of sanitation =improved				***Ref.***						***Ref.***		
Wealth Index(quintiles)=Poorest				2.45	2.07, 2.90	0.000				3.35	3.00, 3.74	0.000
Wealth Index(quintiles)=Second				1.98	1.70, 2.31	0.000				2.65	2.41, 2.93	0.000
Wealth Index(quintiles)=Middle				1.39	1.20, 1.62	0.000				1.81	1.65, 2.00	0.000
Wealth Index(quintiles)=Fourth				1.13	0.97, 1.31	0.104				1.47	1.35, 1.61	0.000
Wealth Index(quintiles)=Richest				***Ref.***						***Ref.***		

BM=Breast Milk ; CF=Complementary feeding; NHM=Non-human milk(formula+animal milk)

Model 1: Feeding exposure only, adjusted for child age and survey round

Model 2: Estimates are adjusted for area and household parameters.
All estimates are adjusted for child age and survey round and account for the complex survey design (stratification, clustering and rescaled sampling weights)

Table S6. Association between feeding category and wasting among children aged 6–23 months: survey-weighted logistic regression

	Wasting: 6-11 month						Wasting: 12-23 month					
	Model-1			Model-2			Model-1			Model-2		
	**OR**	**95% CI**	**p-value**	**OR**	**95% CI**	**p-value**	**OR**	**95% CI**	**p-value**	**OR**	**95% CI**	**p-value**
Feeding=CF	0.92	0.60, 1.41	0.692	1.18	0.72, 1.93	0.522	0.93	0.81, 1.06	0.264	0.90	0.78, 1.04	0.145
Feeding=NHM	1.67	1.37, 2.04	0.000	1.99	1.59, 2.48	0.000	0.99	0.82, 1.18	0.877	1.12	0.92, 1.35	0.251
Feeding=NHM+CF	1.14	0.95, 1.35	0.151	1.34	1.12, 1.61	0.001	0.75	0.69, 0.81	0.000	0.80	0.74, 0.87	0.000
Feeding=BM	1.14	1.01, 1.30	0.036	1.09	0.95, 1.26	0.221	1.05	0.93, 1.17	0.439	1.00	0.88, 1.14	0.972
Feeding=BM+CF	***Ref.***			***Ref.***			***Ref.***			***Ref.***		
Feeding=BM+NHM	1.21	1.04, 1.40	0.012	1.24	1.05, 1.45	0.010	1.09	0.96, 1.24	0.171	1.12	0.97, 1.30	0.128
Feeding=BM+NHM+CF	1.11	0.98, 1.26	0.101	1.20	1.05, 1.37	0.006	0.93	0.86, 1.00	0.062	0.97	0.89, 1.05	0.387
Region=Urban				***Ref.***						***Ref.***		
Region=Rural				0.87	0.78, 0.98	0.020				0.85	0.78, 0.94	0.001
Province=Punjab				***Ref.***						***Ref.***		
Province=Sindh				1.28	1.12, 1.46	0.000				1.20	1.09, 1.31	0.000
Province=KP				1.26	1.08, 1.46	0.003				0.81	0.72, 0.91	0.001
Province=Balochistan				0.81	0.66, 1.00	0.046				0.78	0.68, 0.90	0.001
Main Source of drinking water=unimproved				1.01	0.83, 1.22	0.959				0.97	0.85, 1.12	0.715
Main Source of drinking water=improved				***Ref.***						***Ref.***		
Main Source of sanitation =unimproved				1.17	1.03, 1.32	0.013				1.23	1.14, 1.33	0.000
Main Source of sanitation =improved				***Ref.***						***Ref.***		
Wealth Index(quintiles)=Poorest				1.74	1.43, 2.12	0.000				1.69	1.44, 1.97	0.000
Wealth Index(quintiles)=Second				1.63	1.38, 1.92	0.000				1.48	1.28, 1.71	0.000
Wealth Index(quintiles)=Middle				1.43	1.22, 1.69	0.000				1.21	1.05, 1.39	0.007
Wealth Index(quintiles)=Fourth				1.21	1.03, 1.43	0.018				1.11	0.99, 1.26	0.078

Wealth Index(quintiles)=Richest				***Ref.***						***Ref.***		

BM=Breast Milk ; CF=Complementary feeding; NHM=Non-human milk(formula+animal milk)

Model 1: Feeding exposure only, adjusted for child age and survey round
Model 2: Estimates are adjusted for area and household parameters.

All estimates are adjusted for child age and survey round and account for the complex survey design (stratification, clustering and rescaled sampling weights)

Table S7. Association between feeding category and multiple micronutrient deficiencies among children aged 6–23 months: survey-weighted logistic regression

	MMN: 6-11 month						MMN: 12-23 month					
	Model 1			Model 2			Model 1			Model 2		
	**OR**	**95% CI**	**p-value**	**OR**	**95% CI**	**p-value**	**OR**	**95% CI**	**p-value**	**OR**	**95% CI**	**p-value**
Feeding=CF	1.03	0.35, 3.07	0.952	1.32	0.44, 3.97	0.626	0.67	0.46, 0.97	0.035	0.71	0.49, 1.03	0.071
Feeding=NHM	1.34	0.79, 2.27	0.270	1.32	0.78, 2.25	0.304	1.06	0.68, 1.68	0.787	0.97	0.61, 1.53	0.886
Feeding=NHM+CF	1.32	0.85, 2.05	0.216	1.31	0.83, 2.05	0.247	1.37	1.10, 1.72	0.005	1.28	1.03, 1.61	0.029
Feeding=BM	0.84	0.61, 1.16	0.295	0.87	0.62, 1.21	0.400	0.91	0.70, 1.18	0.478	0.91	0.70, 1.19	0.496
Feeding=BM+CF	***Ref.***			***Ref.***			***Ref.***			***Ref.***		
Feeding=BM+NHM	0.85	0.57, 1.27	0.436	0.81	0.54, 1.22	0.311	1.54	1.08, 2.21	0.018	1.50	1.05, 2.13	0.026
Feeding=BM+NHM+CF	1.23	0.88, 1.71	0.220	1.21	0.86, 1.69	0.267	1.20	0.98, 1.46	0.073	1.15	0.94, 1.41	0.188
Region=Urban				***Ref.***						***Ref.***		
Region=Rural				0.92	0.68, 1.25	0.603				0.88	0.73, 1.07	0.193
Province=Punjab				***Ref.***						***Ref.***		
Province=Sindh				0.92	0.69, 1.22	0.557				0.74	0.61, 0.91	0.004
Province=KP				0.70	0.45, 1.08	0.109				0.72	0.55, 0.93	0.013
Province=Balochistan				0.55	0.33, 0.92	0.023				0.63	0.46, 0.86	0.004
Main Source of drinking water=unimproved				1.02	0.63, 1.66	0.935				0.62	0.41, 0.94	0.025
Main Source of drinking water=improved				***Ref.***						***Ref.***		
Main Source of sanitation =unimproved				1.00	0.70, 1.45	0.987				0.98	0.75, 1.29	0.912

	MMN: 6-11 month						MMN: 12-23 month					
	Model 1			Model 2			Model 1			Model 2		
	**OR**	**95% CI**	**p-value**	**OR**	**95% CI**	**p-value**	**OR**	**95% CI**	**p-value**	**OR**	**95% CI**	**p-value**
Main Source of sanitation =improved				***Ref.***						***Ref.***		
Wealth Index(quintiles)=Poorest				1.35	0.85, 2.13	0.200				1.07	0.76, 1.50	0.712
Wealth Index(quintiles)=Second				1.12	0.74, 1.68	0.602				0.98	0.73, 1.31	0.893
Wealth Index(quintiles)=Middle				0.93	0.63, 1.37	0.718				0.92	0.71, 1.18	0.502
Wealth Index(quintiles)=Fourth				1.01	0.70, 1.46	0.955				1.06	0.83, 1.36	0.636
Wealth Index(quintiles)=Richest				***Ref.***						***Ref.***		

BM=Breast Milk ; CF=Complementary feeding; NHM=Non-human milk(formula+animal milk)

Model 1: Feeding exposure only, adjusted for child age and survey round
Model 2: Estimates are adjusted for area and household parameters.

All estimates are adjusted for child age and survey round and account for the complex survey design (stratification, clustering and rescaled sampling weights)

Table S8: Unadjusted and adjusted p-values for model 3 for stunting outcome

	Age: 6-11 month		Age :12-23 month	
	P-values			
	Unadjusted	Adjusted	Unadjusted	Bonferroni-adjusted
Feeding=CF	0.836	0.999	0.01	0.03
Feeding=NHM	0.001	0.003	0.418	0.418
Feeding=NHM+CF	0.375	0.5625	<0.0001	<0.0006
Feeding=BM	<0.0001	0.0006	0.102	0.204
Feeding=BM+CF	Ref	Ref	***Ref.***	
Feeding=BM+NHM	0.023	0.046	0.387	0.464
Feeding=BM+NHM+CF	0.841	0.841	0.341	0.409

p-values are adjusted for Benjamini–Hochberg false discovery rate
